# A Feedfordward Adaptive Controller to Reduce the Imaging Time of Large-Sized Biological Samples with a SPM-Based Multiprobe Station

**DOI:** 10.3390/s120100686

**Published:** 2012-01-10

**Authors:** Jorge Otero, Hector Guerrero, Laura Gonzalez, Manel Puig-Vidal

**Affiliations:** SIC-BIO, Bioelectronics and Nanobioengineering Group, Department of Electronics, University of Barcelona, Marti i Franques, 1, 08028, Barcelona, Spain; E-Mails: hguerrero@el.ub.es (H.G.); lgonzalez@el.ub.es (L.G.); manel.puig@ub.edu (M.P.-V.)

**Keywords:** scanning probe microscopy, feedforward control, adaptive control, nanorobotics, fast imaging, biological samples, bacteria

## Abstract

The time required to image large samples is an important limiting factor in SPM-based systems. In multiprobe setups, especially when working with biological samples, this drawback can make impossible to conduct certain experiments. In this work, we present a feedfordward controller based on bang-bang and adaptive controls. The controls are based in the difference between the maximum speeds that can be used for imaging depending on the flatness of the sample zone. Topographic images of *Escherichia coli* bacteria samples were acquired using the implemented controllers. Results show that to go faster in the flat zones, rather than using a constant scanning speed for the whole image, speeds up the imaging process of large samples by up to a 4× factor.

## 1. Introduction

Life sciences studies using Scanning Probe Microscopy (SPM) have been growing incredibly in the last decades and, specially, Atomic Force Microscopy (AFM) is the basis of several research works in both molecular and cell biology. AFM is based on the measurement of the force interactions between a nanometric tip and a sample surface. Due to the specific characteristics of the technology, it allows researchers to study the nanoscale properties of cells and biomolecules under physiological conditions at unprecedented level of detail. Results reported in the last years about morphological [1], mechanical [2], electrical [3] and chemical properties of biological samples have revealed extremely important information about the behavior, structure and dynamical processes occurring in cells [4], bacteria [5] and biomolecules [6]. These results could be the basis in the near future for prognosis and treatment of cancer [7], fighting infectious diseases [8], molecular medicine [9], and several other important topics in the biomedical field.

However, in biological studies, AFM still presents some limitations. Two of the main ones are the use of only one probe to measure different properties of the sample, and the slow imaging rate. In previous works [10,11] the authors of this paper have developed a multiprobe station to overcome the first limitation, as have done others [12,13], but these solutions for the first problem increase the duration of experiments even more; they hinder the imaging of dynamical processes and the conduction of long experiments with some biological samples because these become degraded [14]. The slow imaging rate problem has been partially solved for small-sized samples, such as biomolecules, by using a wide range of solutions reported for videoAFM operation [15–17], but imaging time is still one of the main limitations when conducting experiments with cells and bacteria under physiological conditions. The sample is dynamically changing and a large time gap between consecutive steps of the experiment drastically reduces the success rate and can give wrong results and artifacts [18]. In multiprobe experiments, where each probe is devoted to one task, a typical experiment is: the first probe images a wide area of the sample, and the second one performs a mechanical test over the desired location. In these experiments, the imaging process time should be reduced as much as possible to avoid the problems related to the sample degradation time and drift.

Several solutions have been implemented to reduce the AFM image acquisition time, mostly centered on videoAFM applications and involving optimization of the different hardware elements which are responsible of the limits in the speed of operation. Development of high resonant frequency cantilevers, high bandwidth photodetectors, faster scanners [19] and high-speed electronics for data acquisition and control are mainly the approaches to increase the imaging speed from the hardware point of view. Software solutions have also been reported that are focused on the improvement of the response time in the feedback loop. They use different control strategies [20] to speed up the scanning operation by actively controlling its dynamic response [21]. Although all the above solutions are successfully applied for studying very fast processes in biomolecules [22], they are not often applicable to large biological samples, where the areas to scan are in the range of tenths of microns and heights of the samples are in the micron range; for these scenarios, different control strategies are needed [23].

In this work we investigate feedforward control strategies to reduce the image acquisition time in large-sized biological samples. The main idea is to scan faster in the flatter zones and slower in the zones where there is important topographic information. The feedforward control is based on the prediction of the slope of the next sample point during the scanning process. Bang-bang and adaptive controllers are then implemented to adjust the scan speed based on the output of the predictive algorithm. Finally, experiments to show the reduction of the time required to image an average-filled *Escherichia coli* bacteria sample with the multiprobe station are presented.

## 2. Multiprobe Station

**Figure 1.**
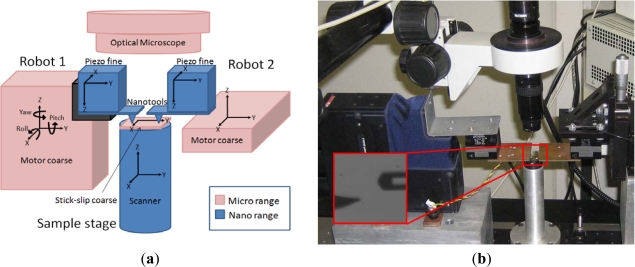
The multiprobe SPM-based nanocharacterization station presented in [10,11,24]. (**a**) Scheme of the different micro and nanopositioning stages. (**b**) Photograph of the developed station with a quartz tuning fork probe and an AFM cantilever as nanotools (optical microscope image).

The multiprobe SPM station we have devised is a 20 degrees-of-freedom (DOF) robotized station based on microstepper motors and piezoelectric actuators working in a closed loop (Figure 1). The basics of the station were described in [10] and the coordination strategies in [11,24]. Briefly, two nanoprobes and the sample can be positioned independently in coarse and fine motion. Although there are recent developments in actuators combining long range and nanometer accuracy, they are still in the research phase [25], so in the developed station, positioning of the probes is performed by using a combination of micro- and nanopositioning stages. Micropositioning is performed in an automated way with a resolution of 40 nm and a repeatability of 5 μm for 12 mm of travel by using microstepper motors from Thorlabs (MT3 and APT604 models). Fine movement is controlled with 2-nm resolution and 25-nm accuracy in a 100-μm^3^ volume by using piezoelectric actuators from PI (Nanocube model). The sample scanner is a piezotube from Nanotec Electrónica S.L. (closed-loop large-scanner model) with nm resolution in the X and Y directions and sub-nm in the Z. The sample can also be coarse positioned in the micro range in the X and Y directions by using the stick-slip technique [26]. The robots can be equipped with different tools, depending on the task to be preformed (AFM cantilevers and quartz tuning fork tools [27]). The common reference system for the platform is a high depth-of-field and long working distance optical microscope.

## 3. Theory

### 3.1. Scanning Speed Limits

The scanning speed limits can be determined analytically for a given sample, cantilever and controller specifications. In large-sized samples, the feedback should be able to follow large Z steps avoiding a high tip-sample interaction force, as well as maintain enough resolution for the flatter zones of the sample. Assuming the system is working in dynamic mode (to minimize the tip-sample interaction), the maximum scanning speed *v_s_* is related with the minimum height difference in the sample to be followed by the feedback *Δz* (the resolution in the Z direction), as reported in [28]:

(1) $$v_{s}=f_{\mathrm{bw}}\cdot \Delta z$$

where the maximum scanning speed is limited by the feedback bandwidth *f_bw_*. When large steps appear in the sample, the maximum scan speed *v_s_* can be calculated [29]:

(2) $$v_{s}=\frac{\left(A_{\mathrm{far}}-A_{\mathrm{setp}}\right)\cdot \left(1-e^{\frac{-1}{2Q}}\right)\cdot \text{tan}(\alpha )}{T}$$

where *A_far_* is the amplitude of the cantilever far from the sample, *A_setp_* is the amplitude used as setpoint for the feedback in a period of oscillation *T* for a given cantilever with quality factor *Q* and aperture angle *α* [20]. Both Equations (1) and (2) depend on the local height of the sample. The maximum speed for flat zones is directly related to the expression in Equation (1), and the maximum speed when large steps appear is related to the expression in Equation (2), which is proportional to the reduction of the oscillation amplitude that is directly related to the height of the Z step in the fast direction of the scanning process.

For large samples, such as bacteria, the maximum speed to get the desired resolution is much greater than the maximum speed given by the larger steps in Z. Then, in a conventional AFM setup, the scan speed must be settled to the slower speed; this fact produces a loss of performance in the flatter zones. In the next sections, authors describe the approximations performed to reduce the imaging time based on the difference in the maximum speed not taken into account in the conventional scanning systems (where the complete image is acquired at a constant speed).

### 3.2. Feedforward Control

**Figure 2.**
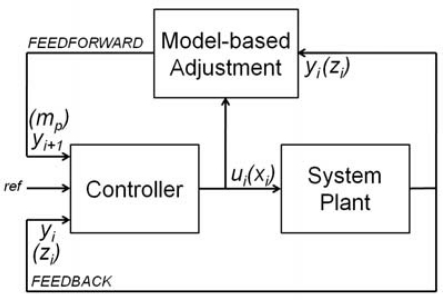
Feedback and feedfordward control scheme. Indexes are the classical nomenclature and the parameters in our system are into brackets.

The most widely used controls in robotics and SPM are based on feedback loops (Figure 2), often based on PID control algorithms: the measurement of the actual state of the system (*y_i_*, the outputs of the plant for a time instant *i*) is used to correct the outputs of the controller (*u_i_*, the inputs of the system). In contrast, a feedforward controllers predicts the future state of the system *y_i+1_* from the actual and past system’s states (*y_i_, y_i_*_−_*_1_, y_i_*_−_*_2_*...) to correct the outputs of the controller.

The main idea in model-based feedforward control (Figure 2) is to update the parameters of the closed loop control in function of the changes in the response of the system plant. In our case, the parameters are the maximum scan speed and the sample slope. The goal is that the speed converge to the ideal value for the predicted height of the sample.

Feedforward control can improve the accuracy of the control by as much as an order of magnitude due to the anticipation of the plant changes and faster response: it mainly depends on the accuracy of the model used to predict the following outputs of the plant. Linear or linearized models are the simplest but most effective to implement feedforward control in robot trajectories [30].

**Figure 3.**
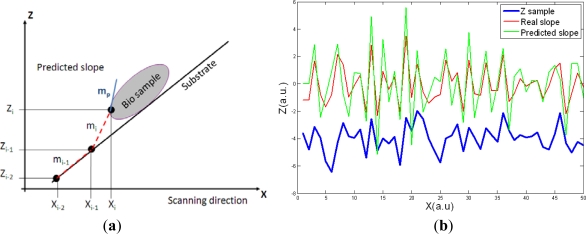
(**a**) The feedforward control is based on predicting the following slope in the sample (*m_p_*) by computing the last two slopes in the sample. (**b**) Comparison between the real and the predicted slope for a randomly generated scan line. The predictive algorithm slightly overestimates the future slope but this fact prevents tip or sample damaging in the experiments.

As the maximum scan speed is related with the Z height, a linear model on the future slope of the sample can be implemented to perform a feedforward control over the scan speed in the X direction. The adjustment mechanism computes the last three heights measured in the sample to calculate the two last slopes. Then, a linear assumption is made in the change in the slope (it will be maintained in the next point in X direction):

(3) $$m_{p}=m_{i+1}=m_{i}-m_{i-1}=\frac{z_{i}-z_{i-1}}{x_{i}-x_{i-1}}-\frac{z_{i-1}-z_{i-2}}{x_{i-1}-x_{i-2}}$$

where *m_p_* is the predicted slope, *m_i_* are the computed previous slopes, *z_i_* are the previous measured heights and *x_i_* are the positions of the scanner in the X direction. The principle of the predictive algorithm is shown in Figure 3(a).

Given the two last slopes appeared in the Z direction, the algorithm predicts the following slope mp. Then, the next X point where feedback should be applied to respond to the slope can be determined for a given scanning speed. In Figure 3(b) the predicted slope and the real slope for a randomly generated sample are presented. The predictive model slightly overestimates the following slope; this will make the control sub-optimal but it will prevent damaging the tip or the sample due to an underestimation of the following slope in the sample.

## 4. Controllers Design

An exact calculation of the optimum speed could be done from the output of the predictive algorithm, but this method would have a high computational cost. In the following subsections, a bang-bang controller and an exponential adaptive controller are presented to adjust the scan speed from the predicted slope.

### 4.1. Bang-Bang Controller

**Figure 4.**
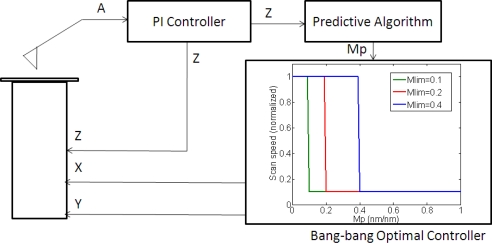
Bang-bang optimal control implementation. *A* is the cantilever oscillation amplitude; X, Y and Z are the scanner driving signals; *m_p_* is the predicted slope.

Once the following slope is predicted by the previously presented algorithm, a bang-bang controller [31] was implemented as a first approach to adjust the scan speed. The controller switched between the maximum scanning speed for flat zones and the minimum scanning speed for regions with sample information. While the predicted slope was lower than a limit value (determined experimentally to avoid sample or tip damage) the speed was set to the maximum speed, assuming that the system was scanning a flat zone; when there was a high predicted slope (higher than the limit value) the speed was switched to the minimum one, which ensured that the system responded to the maximum height steps in the sample (Figure 4).

The limit in the slope where the scan speed was changed was determined experimentally. Then, in the zones where slope was significant, the image was acquired at the same speed than if it were performed using a standard controller. But in the flat zones, the speed was increased to the maximum one. The main benefit of the bang-bang controller was the fast response time because of the low computational cost of the algorithm. However, the controller presented some drawbacks which difficult its use with large biological samples and limits its performance:

- In real experiments, the maximum scan speed had to be set lower than the theoretical maximum because switching between two very different speeds make the system unstable due to the oscillations in the piezoelectric actuators when big accelerations or decelerations occur.
- The limit slope to switch towards the slow speed had to be set very low to avoid damaging the tip/sample.
- Related with the first drawback, oscillations appeared when switching between very different speeds and the predictive algorithm gave erroneous values for the following slope: in the experiment, this was evidenced by the controller: it selected the slow speed most of the time, even in the flat zones.

### 4.2. Adaptive Controller

**Figure 5.**
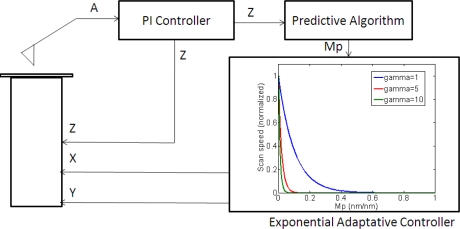
Exponential adaptive controller implementation. A is the cantilever oscillation amplitude; X, Y and Z are the scanner driving signals; *m_p_* is the predicted slope.

The main problem observed with the bang-bang controller was that high accelerations and decelerations caused instabilities and oscillations, limiting the performance of the solution. Then, a possible solution was to make the speed change in a more progressive way. So, the solution was based on the implementation of an adaptive controller [32]. The controller adjusted the scan speed by using the following calculation:

(4) $$v_{\text{scan}}=v_{\text{max}}\cdot e^{-\gamma \cdot \left|m_{p}\right|}$$

where *v_scan_* is the adjustment of the scan speed in the X direction, *v_max_* is the maximum speed as calculated using Equation (1), *m_p_* is the following predicted slope and *γ* is an experimentally tuned attenuation factor. A control scheme is presented in Figure 5.

Depending on the attenuation parameter, the controller had a response which varied from a response closer to the bang-bang controller (high *γ*) to a quasi-linear response (very low *γ*). As it will be presented in the experimental section, the lower the attenuation parameter, the higher the speed in obtaining an image. This parameter had to be tuned experimentally towards a lower *γ* value (by increasing the scan speed), taking care to not to significantly lose image resolution.

**Figure 6.**
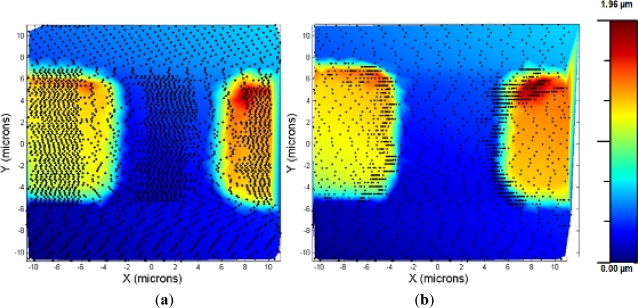
(**a**) Calibration grid imaged using the bang-bang controller, imaging time = 1 min 22 s. (**b**) Calibration grid imaged using the adaptive controller, imaging time = 52 s.

An experiment was made to compare the performances between the bang-bang and the adaptive controller: a square calibration grid was imaged using both controllers. As shown in Figure 6, the bang-bang controller was very sensitive to the oscillations produced by the fast speed changes, and the speed was switched to low even in the flat zones of the lines where the grid was imaged. In contrast, the adaptive controller, due to the progressive changing of the scan speed, avoided those problems and imaged the sample faster and the acquisition points were placed in the interest zones of the sample (the ones where a high change in the topography is evidenced).

## 5. Experiments

### 5.1. Experimental Setup

**Figure 7.**
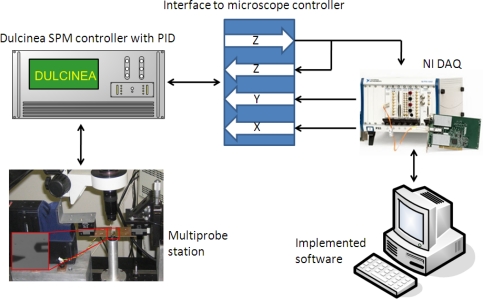
Experimental setup. The controllers were implemented in software; an I/O board was used as interface with the analog control of the SPM controller. The main PID control was executed in the SPM controller.

Images to test the implemented control systems were obtained with the previously presented multiprobe station. The PID control was executed with a Nanotec Dulcinea Controller (10 kHz bandwidth). The predictive algorithm and the bang-bang/adaptive controller were fully implemented in software on a personal computer with a I/O board (DAQ from National Instruments), which was also used for data acquisition (Figure 7). Images were obtained in dynamic mode in ambient conditions. The cantilevers used were rectangular silicon cantilevers from MicroMasch with *K_nom_* = 42 N/m.

The biological sample used was an inoculated culture of *Escherichia coli* dropped over a flat gold surface; in this sample, the maximum Z step were 1 μm and the desired resolution was 5 nm (minimum height of the bacterial appendixes). Resonant frequency and *Q* factor of the cantilever were measured with a lock-in amplifier to be 322.25 kHz and 312 respectively. Then, the maximum and minimum speeds for this sample were 50 μm/s and 2.98 μm/s, respectively, obtained by using the expressions presented in Equations (1) and (2).

### 5.2. Bacteria Imaging Using the Bang-Bang Controller

**Figure 8.**
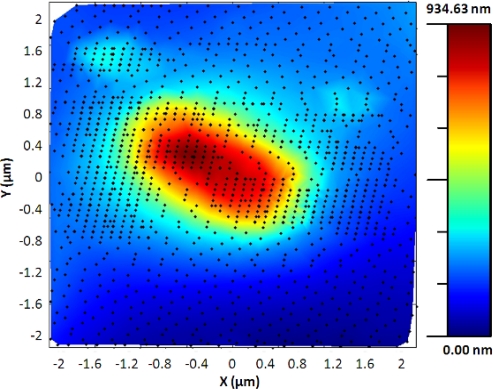
Image acquired with the bang-bang controller of an *Escherichia coli* bacterium. *v_max_* = 8 μm/s, *v_min_* = 2 μm/s, imaging time = 1 min 41 s.

In order to test the bang-bang controller, the output of the predictive algorithm was used as the input of the controller. The image of a single bacterium presented in Figure 8 shows that the controller was very sensitive on the roughness of the sample and the oscillations produced by the fast changes in the scanning speed, and switched to the minimum scan speed most of the time, even in the flat zones of the sample.

The minimum and maximum scan speed needed to be set very low and there was no significant improvement with respect to the standard imaging procedure, no matter whether the X lines were flat or with high Z steps. With the bang-bang approach the improvement with respect to standard AFM imaging (64 lines, 2 μm/s, imaging time of 2 min 8 s) was modest.

### 5.3. Bacteria Imaging Using the Adaptive Controller

**Figure 9.**
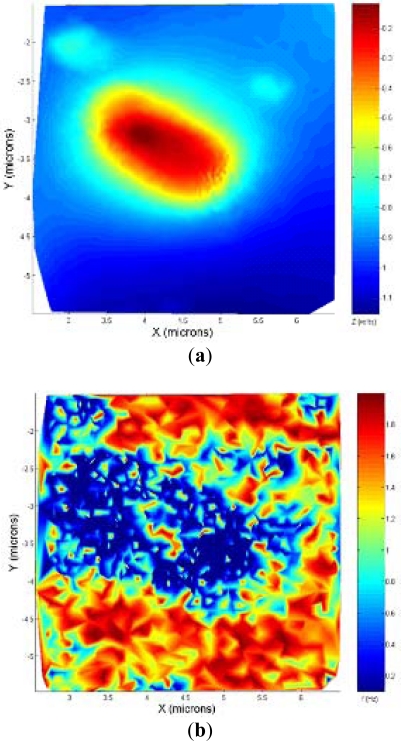
(**a**) Single bacterium imaged using the adaptive controller. (**b**) Frequencies of the scanning signal in the X direction. *γ* = 5, imaging time = 1 min 9 s.

**Figure 10.**
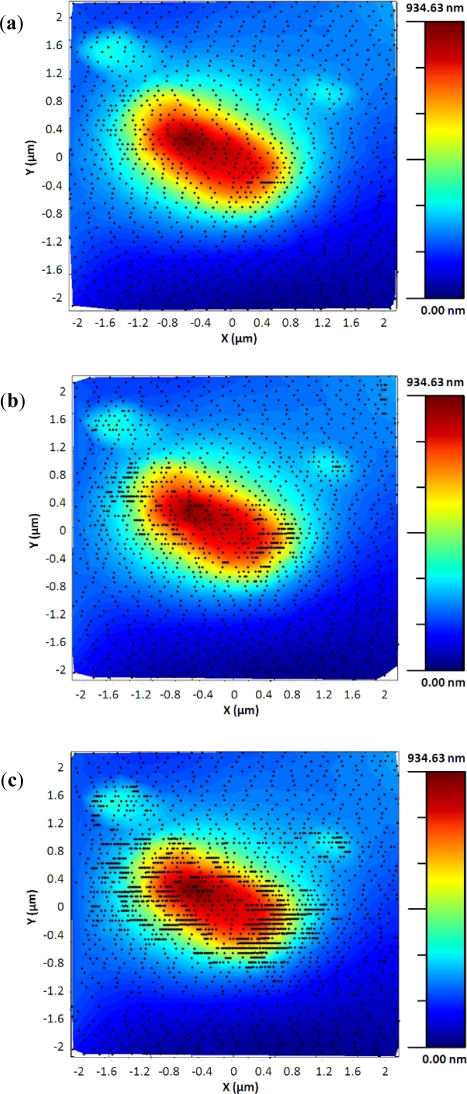

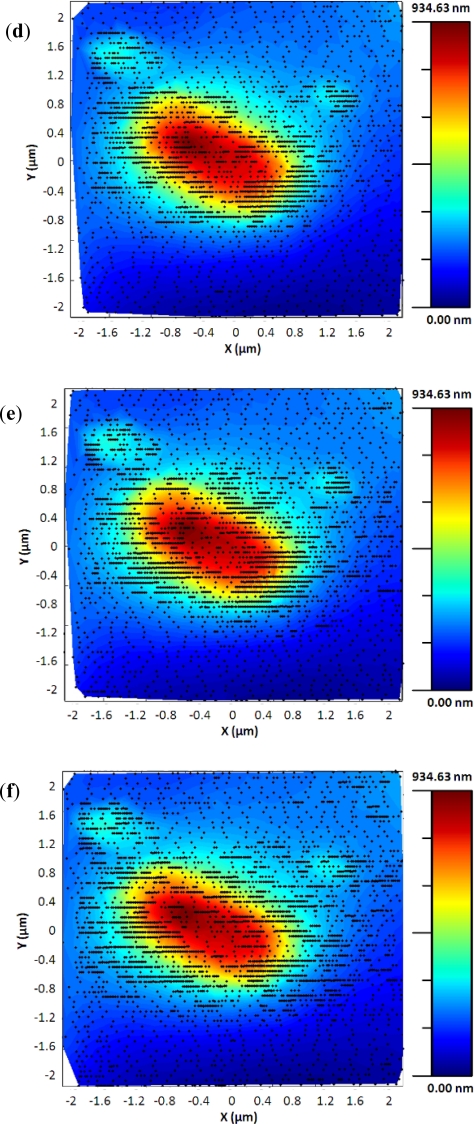
Tests of the adaptive controller over the same bacteria sample zone for different attenuation factors. (**a**) *γ* = 1, imaging time = 36 s. (**b**) *γ* = 2, imaging time = 43 s. (**c**) *γ* = 4, imaging time = 1 min 1 s. (**d**) *γ* = 6, imaging time = 1 min 13 s. (**e**) *γ* = 8, imaging time = 1 min 21 s. (**f**) *γ* = 10, imaging time = 1 min 27 s.

The adaptive controller response was slightly slower than the bang-bang one but it presented an increase of resolution in the zones of interest. This was because the exponential adaptive controller was designed to take into account small flat zones in complex scenarios. The scan speed was adapted in function of the predicted slope following an exponential law. A map of the imaging scanning frequencies for a single bacterium is presented in Figure 9. Figure 10 shows the results obtained for different damping factors (*γ*) in the algorithm over the same sample zone (Imaging time by using standard AFM with the speed settled to 2 μm/s would be 2 min 8 s).

As expected, increasing the attenuation factor produced images with more resolution but increased the imaging time. Meanwhile, low γ produced images with an important lack of resolution. For this kind of sample, no significant change in resolution was observed for attenuation factors greater than 6, so this value were determined as the optimal one under these concrete experimental conditions.

**Figure 11.**
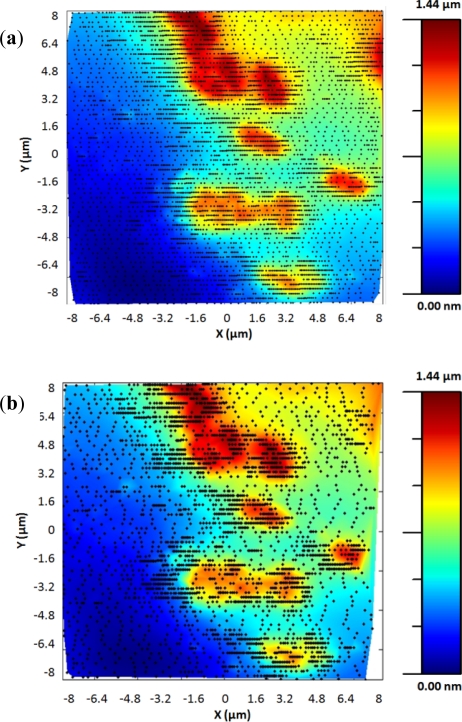

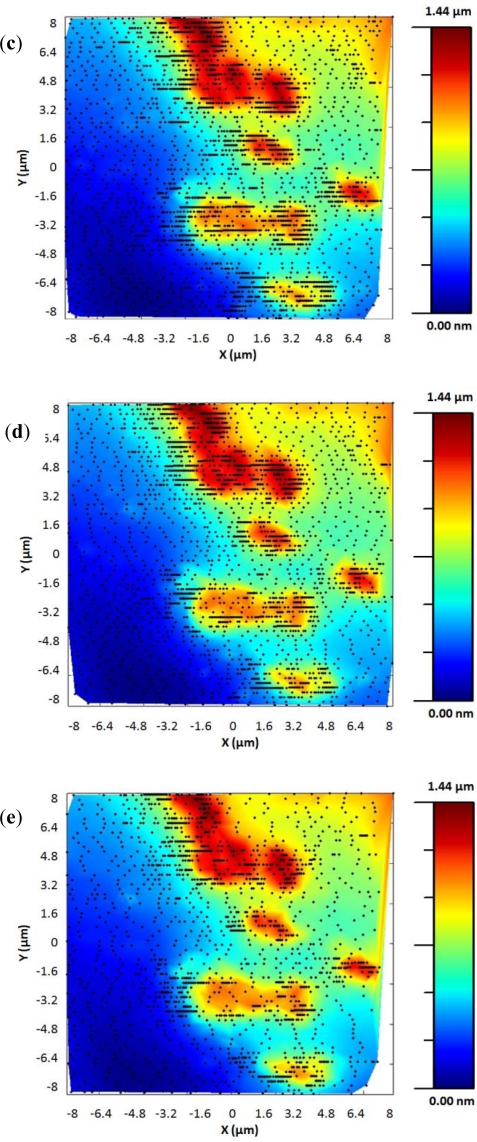

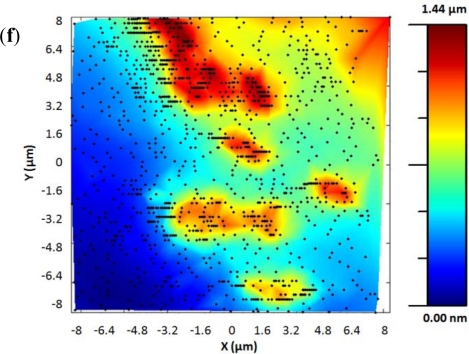
Tune of the different parameters. (**a**) *γ* = 8, *v_max_* = 16 μm/s, imaging time = 2 min 23 s. (**b**) *γ* = 8, *v_max_* = 32 μm/s, imaging time = 1 min 21 s. (**c**) *γ* = 6, *v_max_* = 32 μm/s, imaging time = 1 min 11 s. (**d**) *γ* = 5, *v_max_* = 32 μm/s, imaging time = 1 min 6 s. (**e**) *γ* = 5, *v_max_* = 48 μm/s, imaging time = 45 s. (**f**) *γ* = 5, *v_max_* = 64 μm/s imaging time = 34 s.

The maximum speed can be calculated theoretically if the height of the maximum Z step in the sample is known. Because of it is very difficult to predict the slopes in biological samples (*i.e.*, there could be two bacteria one over the other), the strategy to set the parameters of the controller is shown in Figure 11. The procedure to tune the algorithm was the following:

1. Select the minimum attenuation factor which produced images with enough resolution
2. With the selected attenuation factor, test different maximum scan speeds.
3. Once the maximum scan speed was determined, the attenuation factor was re-tuned if needed.

We started with a high *γ* factor (8) and a low scan speed. Progressively the *γ* factor was lowered and the scan speed increased. Finally, the tuned parameters, for this sample, were *γ* factor of 5–6 and scan speed of 32–48 μm/s because they produced similar images with similar acquisition times.

**Figure 12.**
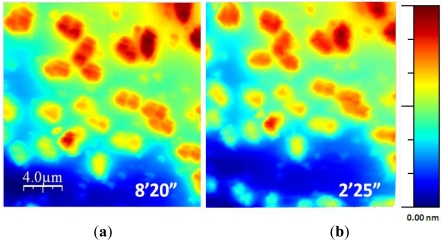
(**a**) *Escherichia coli* bacteria imaged with a standard technique, and (**b**) the implemented adaptive control. The imaging time has been reduced to the 29%.

Finally, Figure 12 shows two topographic images of the same sample. They were acquired using the standard procedure and the exponential adaptive controller. The second image was acquired using the adaptive controller with a *γ* of 6 and maximum scan speed of 32 μm/s in a time of 2 min and 25 s, related to the 8 min and 20 s needed using the standard procedure. The new control method improved the acquisition time approximately in a 4× factor.

## 6. Conclusions

A new control strategy to speed up the imaging process was presented. It was used to measure the topography of *Escherichia coli* bacteria. Results show that the implemented exponential adaptive controller is able to speed up the imaging process for large samples. The improvement that it represents with respect the classical control depends on the sample characteristics: the more flat zones in the sample the faster the image is acquired because the controller adapts the scan speed in function of the local heights of the sample. For a “medium” sample (about 50% flat, 50% bacteria) the developed controller speeds up the imaging process by a 4× factor, while maintaining the resolution of the image in the zones of interest. Furthermore, it is fully implemented in software, so it can be used not only in the developed multiprobe station, but it can also be integrated in any SPM system with external scanning control.

One of the most important points from the technological point of view is that the speed up of the imaging process does not require changes in the hardware of the SPM system. The improvement is purely on the control side and any SPM system could potentially benefit from the development. On the other hand, improvements in the instrument hardware, like using a higher bandwidth piezoelectric actuator for the scanning process, will also be compatible with the developed adaptive control and images will be acquired even faster.

The reduction of the imaging time by speeding the scanning process in the flat zones is very important in multiprobe applications: reduction of the imaging time is critical for avoiding drift between the different probes. Also, when working with biological samples, it will allow the realization of more complex experiments which are now limited by two main factors: the first one is the fact that samples change quicker than the time needed to acquire an image with the first probe and interchange the probes’ position; the second limitation is that samples often become degraded before the experiment (which usually requires the acquisition of several images) is finished.
